# First line chemotherapy plus trastuzumab in metastatic breast cancer HER2 positive - Observational institutional study

**DOI:** 10.11604/pamj.2016.24.324.4058

**Published:** 2016-08-24

**Authors:** Meryem Aitelhaj, Siham Lkhoyaali, Ghizlane Rais, Saber Boutayeb, Hassan Errihani

**Affiliations:** 1 Medical Oncology Department, National Institute of Oncology, Rabat, Morocco

**Keywords:** HER-2 metastatic breast cancer, trastuzumab

## Abstract

Breast cancer is the most common malignant disease and among the most frequent causes of cancer mortality in females worldwide. Metastatic breast cancer (MBC) is conventionally considered to be incurable. In first-line treatment of HER-2 positive MBC, randomized trials have demonstrated that trastuzumab when combined with chemotherapy significantly improves progression free survival and overall survival. To evaluate survival and toxicity of chemotherapy with Trastuzumab as first line therapy of human epithermal growth factor receptor 2 positive metastatic breast cancer, in Moroccan population. It is a phase IV observational institutional monocentric study. Including patients with metastatic breast cancer HER2 positive, as first-line chemotherapy combined with Trastuzumab from March 2009 until March 2010. Primary end point: progression free survival, secondary end point response rate and overall survival. A total of 20 patients were enrolled between March 2009 and March 2010. The lung was the first metastatic site in 60% of the cases, followed by bone, liver, nodes, skin and brain. All patients received chemotherapy with Trastuzumab: 9 of them with Docetaxel, 8 with vinorelbine, and 3 with capecitabine. The progression free survival was estimated by the Kaplan-Meier method, from the date of first cycle to the date of progression or at the last consultation, and the median was 12.8 months. Trastuzumab based chemotherapy was generally well tolerated; 5 patients (25%) presented cardiotoxicity. The results of this study join the literature and show the benefit of Trastuzumab to chemotherapy in first line metastatic breast cancer HER-2 positive.

## Introduction

Metastatic breast cancer (MBC) is conventionally considered to be incurable, with median survival estimates to be in the range of 3 years [1]. Overexpression of human epidermal growth factor receptor type 2 (HER2) occurs in 20-25% of invasive breast cancers, which is associated with a poor prognosis, shortened disease-free interval and high mortality rate [2, 3]. Trastuzumab is a monoclonal antibody targeting the external domain of the c-erb-2 receptor. In first-line treatment of HER2-positive MBC, randomized trials have demonstrated that trastuzumab when combined with paclitaxel or docetaxel significantly improves median time to progression objective response and overall survival [3, 4]. The purpose of this study is to evaluate the efficacy and safety of Trastuzumab plus chemotherapy in metastatic breast cancer HER2 positive in clinical practice in a sample Moroccan population.

## Methods

This is a prospective observational institutional study conducted at the Department of Clinical Oncology, in the national institute of oncology of Rabat from March 2009 to March 2010.

**Eligibility criteria**: Eligible patients had locally advanced or metastatic breast cancer verified histologically and HER2 positive status assessed by immunohistochemistry (3+) or fluorescent in situ hybridization positivity. Ineligibility criteria included HER2 negative status and all patients receiving already first line treatment of MBC with trastuzumab.

**Study design**: The study respected the ethical rules for medical research involving human subjects as stipulated by the World Medical Association in the Declaration of Helsinki. The local ethical committee of the national institute of oncology of Rabat also approved this study; and patients gave their consent. Trastuzumab was administered every 3 weeks with different chemotherapy regimens. Assessment of response was performed using RECIST criteria version 1.1 (Response evaluation criteria in solid tumours). A Progression-free survival (PFS) was calculated from the date of start of treatment until progression or until the date of last visit, overall survival (OS) was calculated from the diagnostic of metastatic disease to the date of last visit. Cardiotoxicity was defined as a left ventricular ejection function decrease below normal values (50%) or an absolute decrease of >10 points or symptoms of heart failure.

**Follow up**: Patients were followed up until January 2014. Any patients who were not reviewed in the last consultation were contacted again by telephone.

**Statistical analysis**: Data was analysed using an electronic CRF (case report form). The information was recorded in an Excel database and analysed with the statistics software SPSS, version 12.0. Descriptive statistics with 95% confidence interval (CI) were calculated according to standard procedure. Survival curves were constructed using the Kaplan-Meier method. A p value less than 0.05 was considered significant.

## Results

A total of 20 patients were enrolled. The median age of patients was 43.5 years. Most of patients had received adjuvant chemotherapy (80%), and all were naive of Trastuzumab. Most of our sample was hormone receptor positive (85%). The number of metastatic site was variable, 50% of patients had one metastatic site and 20% more than 2 sites; the lung was the first metastatic site involved in 60%, followed by bone (50%), liver, nodes, skin and brain. The patient's characteristics are resumed in Table 1. However, it should be stressed that percentages of metastatic sites do not add up to 100% because of overlapping.

**Table 1:** Patient’s characteristics

Characteristic	
**Age**	
median	43.5 ans
Range	30-61
**Hormone receptor status**	
positive	17 (85%)
negative	3 (15%)
HER2 status IHC : 3+	20 (100%)
**Nbre of metastatic sites**	
1	10 (50%)
2	6 (30%)
≥ 3	4(20%)
**Type of metastatic sites**	
Non visceral only	5 (25%)
Viscéral	15 (75%)
Liver	6 (30%)
Lung	12 (60%)
Brain	1 (5%)
Bone	10 (50%)
Skin	2 (10%)
Nodes	6 (30%)
Prior adjuvant chemotherapy	16 (80%)
Prior adjuvant hormonal therapy	14 (70%)
Adjuvant Trastuzumab	0 (0%)

**Treatment delivered** Chemotherapeutic regimens used were different. Nine patients (45%) received Docetaxel (100 mg/m2) every 3 weeks, while 8 other patients (40%) were treated with Vinorelbine (25 mg/m2 at day 1 and 8) every 3 weeks, and 3 patients (15%) with Capecitabine (1000 mg/m2 from day 1 to 14) every three weeks. Trastuzumab was administered every 3 weeks with 8 mg/kg in the first cycle and at subsequent cycles with 6 mg/kg. The median number of chemotherapy cycles received was 9 cycles, and the median of trastuzumab cycles was 15 as shown in Table 2.

**Table 2:** Treatment delivered

**Number of chemotherapy**	
cycles	
median	9
range	2-34
**Number of Trastuzumab**	
cycles	14
Median	2-50
Range	
**Chemotherapy +**	
**Trastuzumab**	
Docetaxel	9 (45%)
Vinorelbine	8 (40%)
Capecitabine	3 (15%)

**Figure 1:**
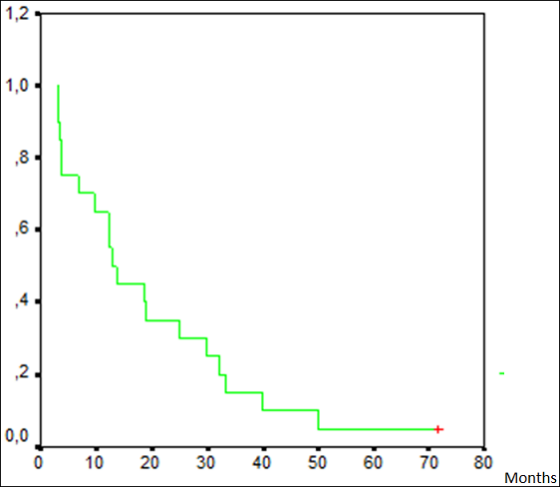
Kaplan Meir curve of progression free survival

**Efficacy:** Most patients had an objective response; 3 of them (15%) achieved complete response, 8 (40%) had partial response and 8 patients (40%) obtained stabilisation with treatment. However for 2 patients (10%), the disease was progressing. (Table 3) The median progression free survival was estimated at 12.8 months as shown in Kaplan Meir curve (Figure 1) and the median overall survival was estimated at 24.5 months.

**Table 3:** Objective response

Type of response	
Complete response	3 (15%)
Clinical benefit	15 (75%)
Partial response	7 (35%)
Stabilisation	8 (40%)
Progression	2 (10%)

**Safety** Trastuzumab based chemotherapy was generally well tolerated; 5 patients (25%) presented cardiotoxicity according to the predefined criteria, 3 (15%) had to discontinue treatment temporarily. In all patients, treatment was resumed after normalising parameters. No patient requires definitive Trastuzumab interruption.

## Discussion

Metastatic breast cancer is conventionally considered to be incurable, with median survival estimates to be in the range of 3 years [1]. Systemic treatment aims to prolong survival, control disease progression, alleviate symptoms, and enhance patient quality of life. HER-2 overexpression occurs in 0-5% of invasive breast cancers and has been shown to play an important role in the pathogenesis of breast cancer and is a marker of aggressive disease and a poor prognosis [2]. Trastuzumab is a recombinant humanized anti-HER-2 monoclonal antibody that targets the extracellular domain of HER-2 with high affinity. Clinical trials have confirmed its value in HER-2-positive MBC as first-line treatment. The pivotal H0648g phase III randomized multinational trial, and the M77001 phae II randomized trial showed respectively that Trastuzumab when combined with Paclitaxel or Docetaxel significantly improves median time to progression (TTP; 7.1 vs 3 with Paclitaxel and 10.6 vs 5.7 with docetaxel), objective response rate (ORR; 49% vs 17% with paclitaxel and 61% vs 34% with Docetaxel) and overall survival (OS; 24.8 vs 17.9 with Paclitaxel and 30.5 vs 22.1 with Docetaxel) [5, 6]. Subsequent trials show benefit of trastuzumab with others chemotherapy drugs such as Vinorlbine, Capecitabine and Gemcitabine; the HERNATA trial confirmed the role of Vinorelbine plus Trastuzumab versus Docetaxel plus Trastuzumab as an alternative first-line therapy combination. In that study, the TTP (median, 12.4 months versus 15.3 months), ORR (59.3% in both arms), and OS time (median, 35.7 months versus 38.8 months) did not differ between arms [7]. The findings of our study were in accordance with the literature data the median TTP was 12.8 months and OS was 24.5 months. Despite this progress the Her2 positive MBC remains a fatal disease. On the other hand, it is very likely that other targeted agents will have a role in the treatment of these patients.

Pertuzumab, a HER-2 targeted monoclonal antibody that prevents HER-2 dimerization, has obtained the FDA approval in first line HER-2 MBC, according to the results of recently published phase III randomized trial (CLEOPATRA study) that assessed Trastuzumab and Docetaxel with either Pertuzumab or placebo demonstrated dramatic results in terms of the PFS time (1.5 months versus 12.4 months). There was a higher response rate (0.2% versus 69.3%) and also a trend toward a longer survival time for the dual HER-2 blockade [8]. Trastuzumab emtansine (T-DM1) is a unique antibody drug conjugate that incorporates the HER-2 targeted antitumor properties of Trastuzumab with the cytotoxic activity of the microtubule-inhibitory agent DM1. T-DM1 allows intracellular drug delivery specifically to HER-2 over expressing cells. Conferring selectivity to the cytotoxic agent and thus increasing the therapeutic index [9]. In the first line setting, T-DM1 was compared with Trastuzumab plus Docetaxel in a phase II study and was associated with high efficacy. A significantly longer PFS interval (14.2 months versus 9.2 months) and greater rate of durable response (median duration, not reached versus 9.5 months), whereas the ORR did not differ between arms [10]. A phase III study of T-DM1 plus Pertuzumab or T-DM1 plus placebo versus Trastuzumab plus a taxane in first line treatment is ongoing [11].

## Conclusion

Despite the small sample of our study we have demonstrated the benefit survival and the good tolerability of Trastuzumab across Moroccan patients. Actually the standard of care in first line treatment for HER2 MBC is the dual HER-2 blockade Pertuzumab associated with Trastuzumab and Docetaxel. However the accessibility and cost of drugs remained a problem in a developing country.

### What is known about this topic

- Trastuzumab widely used in Metastatic Breast cancer;
- Improve progression free survival and overall survival.

### What this study adds

- Evaluate the efficacy and safety of trastuzumab in Moroccan population.
